# Outer retina micro-inflammation is driven by T cell responses prior to retinal degeneration in early age-related macular degeneration

**DOI:** 10.3389/fimmu.2025.1520188

**Published:** 2025-02-05

**Authors:** Lucas Stürzbecher, Hendrik Bartolomaeus, Theda U. P. Bartolomaeus, Sylvia Bolz, Andjela Sekulic, Marius Ueffing, Simon J. Clark, Nadine Reichhart, Sergio Crespo-Garcia, Nicola Wilck, Olaf Strauß

**Affiliations:** ^1^ Experimental Ophthalmology, Department of Ophthalmology, Charité - Universitätsmedizin Berlin, Corporate Member of Freie Universität and Humboldt Universität zu Berlin, Berlin, Germany; ^2^ Experimental and Clinical Research Center, a Cooperation of Charité-Universitätsmedizin Berlin and Max-Delbrück-Center for Molecular Medicine, Berlin, Germany; ^3^ Max-Delbrück-Center for Molecular Medicine in the Helmholtz Association, Berlin, Germany; ^4^ Eye Center, Medical Center, Faculty of Medicine, University Medical Center Freiburg, Freiburg, Germany; ^5^ DZHK (German Centre for Cardiovascular Research), Partner Site Berlin, Berlin, Germany; ^6^ Institute of Experimental Biomedicine, University Hospital Würzburg, Würzburg, Germany; ^7^ Charité-Universitätsmedizin Berlin, Corporate Member of Freie Universität Berlin and Humboldt-Universität zu Berlin, Berlin, Germany; ^8^ Institute for Ophthalmic Research, Department for Ophthalmology, Eberhard Karls University of Tübingen, Tübingen, Germany; ^9^ Department for Ophthalmology, University Eye Clinic, Eberhard Karls University of Tübingen, Tübingen, Germany; ^10^ Lydia Becker Institute of Immunology and Inflammation, Faculty of Biology, Medicine, and Health, University of Manchester, Manchester, United Kingdom; ^11^ École d’optométrie, University of Montreal, Montréal, QC, Canada; ^12^ Department of Nephrology and Internal Intensive Care Medicine, Charité - Universitätsmedizin Berlin, Corporate Member of Freie Universität Berlin and Humboldt-Universität zu Berlin, Berlin, Germany

**Keywords:** age related macular degeneration, geographic atrophy, T cells, adaptive immune system, neurodegeneration

## Abstract

**Introduction:**

Age-related macular degeneration (AMD) is a leading cause of blindness with limited treatment options. Dysfunction of the retinal pigment epithelium (RPE) is a unifying salient feature of the pathology and a primary end-point damage leading to complications such as geographic atrophy (GA), which represents the most common end-stage of AMD.

**Methods:**

Human and murine ocular tissues were used for histological examinations. Furthermore, flow cytometry and gene expression analysis were used on ocular and splenic tissues of *Cx3cr1* ^GFP/GFP^ and *C57BL/6J* mice at 8 and 12 months of age to characterize the dynamics of local and systemic T cell populations.

**Results:**

We show the presence of memory T cells such as CD45RO^+^ cells in the choroid and retina of patients with AMD with a peak of abundance in early stages of AMD. As further evidence for the contribution of the adaptive immune system to GA we identified an increased frequency of CD44^+^ CD69^+^ KLRG1^+^ T cells and para-inflammation of the retina in a mouse model that mimics features of GA. Importantly, the activation of T cells found at early AMD-like stages prior to degeneration possessed long-lasting cytotoxic properties and adopted typical features of senescent immune cells. T cells were intimately associated with the RPE, suggesting transmigration and participating in local micro-inflammation.

**Discussion:**

Our data support that activation and accumulation of memory T cells can be considered as a hallmark of early AMD, and that adaptive immunosenescence likely to contribute to the chronic inflammation associated with RPE damage and the progression to large lesions as seen in GA.

## Introduction

The immune response in its many forms is an intrinsic component of retinal degeneration. While para-inflammation consists of the controlled adaptive response of a tissue to stress, understanding these options in the context of aging is central to maintain and restore retinal homeostasis (1, 2). Some of the known cellular immunological drivers to this process are accumulating resident microglia, infiltrating monocytes, and choroidal macrophages as part of the innate immune response. These reactions are further accompanied by activation of complement factors, cytokines, the inflammasome and other metabolic pathways leading to reactive oxygen species (ROS). The accumulation of such excessive immune responses leads to the manifestation of a tissue-destructive chronic low-grade inflammation (3–5), which appears long before degeneration becomes apparent and can be diagnosed. This para-inflammatory profile is typical in age-dependent macular degeneration (AMD), a major cause for blindness in the elderly population in industrialized countries. In fact, life-long para-inflammation is involved in the progression of AMD and leads to more pervasive end-stages including the geographic atrophy (GA) or neovascular AMD. Understanding the nature and source of this inflammation is paramount to define better strategies to treat patients before retinal damage is beyond repair.

Beyond the participation of mononuclear phagocytes and the innate immune response in AMD (6), a few clinical investigations also confirmed other systemic immune profile alterations in T cell frequencies in AMD patients (7). More specific analyses have delved into T cell accumulation (8) and possible involvement of cytotoxic T cells, Th17 and T cell receptor γδ (TCRgd)-positive cells (9–17). Recently, *Wu* et al. reviewed in detail the current knowledge regarding the adaptive immune landscape in ocular pathologies (18). However, the evidence of the involvement of the adaptive immune system during the pathogenesis of AMD is sparse and lacks a systematic investigation.

As some of these studies hinted at T cell-dependent mechanisms, we hypothesize that T cells and the adaptive immune system participate of the chronic low-grade immune reaction in the outer retina and contribute to processes driven by the innate immune system in AMD that possess factual displays of degeneration. Here, we investigated the occurrence, dynamics, and characteristics of relevant T cell populations using comparative analyses of retinal and systemic immunophenotypes in human post-mortem ocular specimens of patients with AMD and in *Cx3cr1*-deficient mice, a model that reproduces cardinal degenerative features of GA. Starting from 12 months of age, these mice present the following hallmarks relevant to human pathology: drusen-like deposits, retinal microglia accumulation and degeneration of retinal pigment epithelium (RPE), leading to subsequent thinning of photoreceptor cell layers (19–22). Aging in this model does not elicit subretinal neovascularization. Taken together, we present evidence for the activation of the adaptive immune system at 7-8 months, long before the onset of GA-like phenotypic changes.

## Materials and methods

Detailed description of all methods can be found in the **Supplementary Information**.

### Human ocular specimens

Human donor eye tissue was obtained from the Manchester Eye Tissue Repository, an ethically approved Research Tissue Bank (UK NHS Health Research Authority reference no.15/NW/0932). Eye tissue was acquired after the corneas had been removed for transplantation and explicit consent had been obtained to use the remaining tissue for research. Guidelines established in the Human Tissue Act of 2004 (United Kingdom) and the tenets of the Declaration of Helsinki were adhered to. All ocular tissue was processed within 49 hours postmortem and AMD status graded in accordance to the Age-related Eye Disease Study (AREDS) classification. The Manchester Eye Tissue Repository was subsequently transferred to the University of Tübingen and renamed the Helmut Ecker Eye Tissue Resource (HEETR, Tübingen, Germany, local ethical approval 370/2021BO1). We analyzed the ocular globes of patients diagnosed with early (2 patients) or late (3 patients) AMD. In the experiments we used age-and sex-matched controls (3 patients) without any known ocular vascular or inflammatory pathology, nor RPE or retinal lesions. All information with regard of the donors is compiled in **Supplementary Table S1**.

### Animal research

Mice were bred in-house and maintained on a 12-hour light and 12-hour dark cycles, standard environmental conditions and unrestricted access to food and water.

*Cx3cr1* ^GFP/GFP^ mice, hereinafter referred as *Cx3cr1*, were examined at 8 and 12 months of age. *C57BL/6J* mice, hereinafter referred as WT, served as sex- and age-matched controls. Male and female littermates were used in a 1:1 ratio. *Cx3cr1* were bread on a *C57Bl6/J* background. Mice were routinely monitored for mutations in *Crb1* (RD8) and tested negative (23).

At the end of the experiment, mice were anesthetized with isoflurane and euthanized through cervical dislocation. Eyes and spleens were rapidly excised and kept in PBS supplemented with 0.5% BSA (Sigma Aldrich) and 2mM EDTA (Sigma Aldrich) at 4°C. The neurosensory-retina and RPE-choroid complexes were later dissected and analyzed separately depending on the experiment.

### Principal component analysis

We used a principal component analysis (PCA) for dimensional reduction using the *R* package *factoextra* (version 1.0.7). Top 12 features that contributed to the first (Dim. 1) and second (Dim. 2) PCA dimensions were extracted (*stats*, version 3.6.2). For visualization, the plugin *ggplot* was used.

### Statistical analysis

All experiments were repeated at least 3 times. *N* (biological replicates) is indicated in each respective figure legend throughout the manuscript. Values are expressed as the mean ± SD unless indicated otherwise. Statistical analysis was performed using GraphPad Prism 9 and R Studio. In case of single comparisons of two groups, normality was assessed by Kolmogorov-Smirnov test and outliers excluded with Grubb´s test. In groups with n<5 normality could not be tested, and a non-parametric distribution was assumed. Accordingly, groups were tested with two-tailed student´s t-test or two-tailed Mann-Whitney U test depending on the assumption of normal distribution of the data points.

## Results

### Memory T cells accumulate in the human RPE and choroid of patients with early AMD

**Figure 1:**
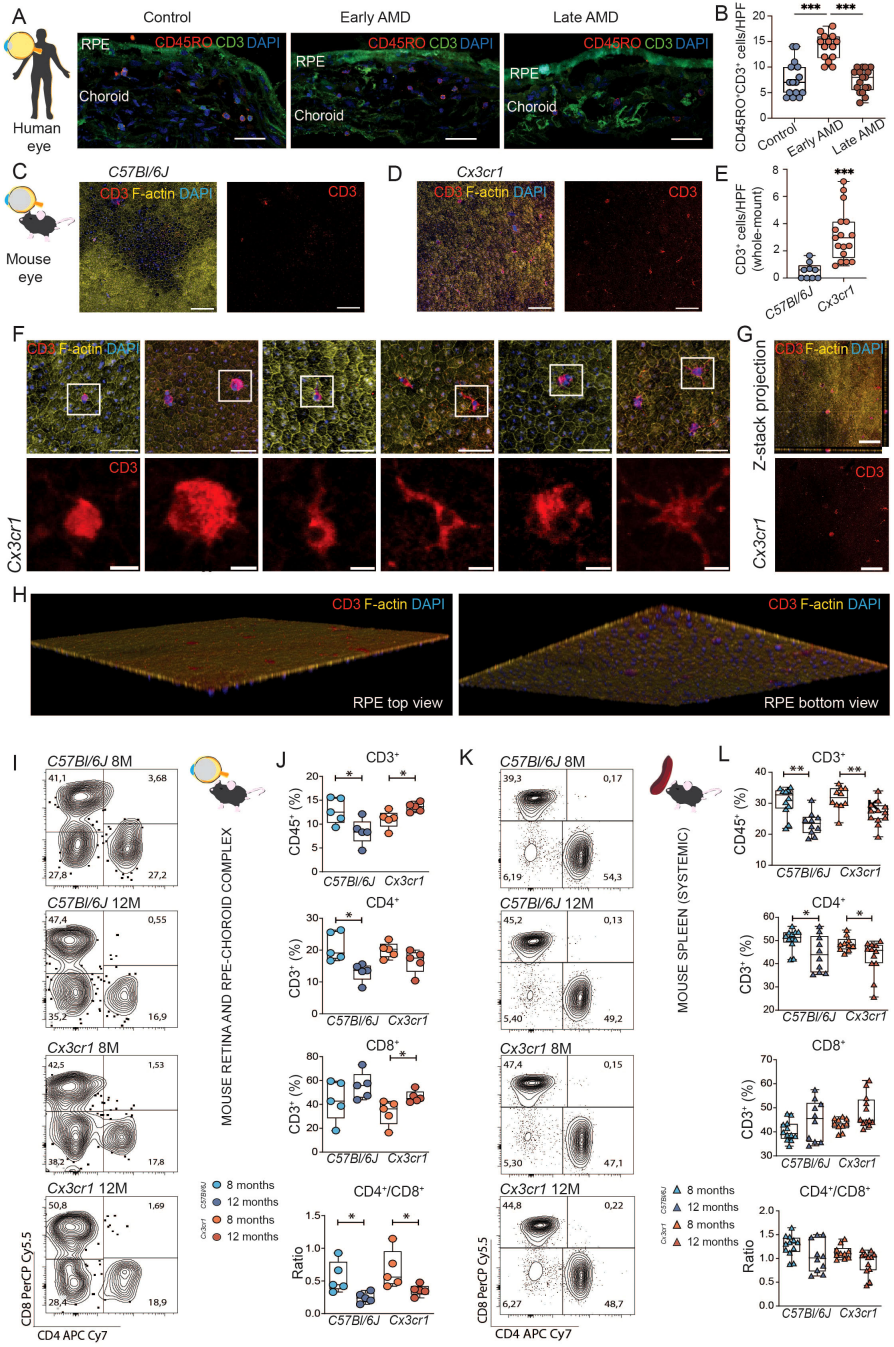
Early retinal T cell accumulation in age-related macular degeneration. **(A)** Histological assessment of retinal T cells in ocular samples from patients with age-related macular degeneration (AMD). Immunofluorescence staining of CD3 and CD45RO. Scale bar 50µm. **(B)** Quantification of CD3^+^ CD45RO^+^ DAPI^+^ memory T cells per high power field (HPF). Data was tested with ordinary one-way ANOVA. ***P < 0,0001. N= 15 HPF from 3 control eyes, N=14 HPF from 2 early AMD eyes, N=17 from 3 late AMD eyes. **(C, D)** Comparison to a mouse model of AMD (*Cx3cr1*). Histological assessment of CD3 T cells in whole mounts of 12-month-old wild type (WT) C57Bl6/J and *Cx3cr1* mice. In *Cx3cr1* T cells are detectable in proximity of central degenerative RPE areas and in mid periphery. Scale bar 100µm. **(E)** Quantification of CD3^+^ DAPI^+^ T cells per high power field (HPF). N= 9 *C57Bl6/J*, N= 17 *Cx3cr1.* **(F)** Morphological variance of T cells in 12-month-old *Cx3cr* whole mounts. Merged scale bar 50µm; CD3 detail scale bar 10µm. Enhanced diameters, amoeboid shaped T cells with filopodia endocytotic and T cells with dendritic morphology are distinguishable. **(G, H)** Three-dimensional reconstruction of the confocal images confirmed that T cells were intimately associated to the basolateral membrane. Left rendering shows apical view, right rendering showing basolateral view. **(I)** Flow cytometric analysis of retinal T cells in *Cx3cr1* and *C57Bl6/J* mice at 8 months and 12 months of age. Representative flow plots for CD4 and CD8 staining within CD45^+^ CD3^+^ T cells. Numbers representing percentage of all included cells into specific gating **(J)** Quantification of CD45^+^ CD3^+^ T cells, CD45^+^ CD3^+^ CD4^+^ CD8^-^ T helper cells, CD45^+^ CD3^+^ CD4^-^ CD8^+^ cytotoxic T cells, and CD4/CD8 ratio. N= 5/group. **(K, L)** same as **(I, J)** but for splenic T cells. N= 10-12/group. All comparisons with Student’s T-test test or Mann-Whitney U test. *p>0.05, ** p>0.01, ***p>0.001.

To determine the functional state of the immune system and the potential presence of T cells in the retina during AMD, we performed histological analyses of the RPE and choroid of patients with early and late AMD compared to age- and sex-matched controls (**Supplementary Table S1**). Overall, we observe a significant increase of memory T cells (CD3^+^CD45RO^+^) in patients with early AMD. These frequencies returned to basal levels at more advanced late AMD stages (**Figures 1A, B**). The findings convey that a recruitment of memory T cells occurs at early stages of AMD and prior to manifesting the degenerative hallmarks of AMD.

### Memory T cells infiltrate the murine retina prior to AMD-like degeneration and show a broad morphological activated-like spectrum

We sought to investigate if the recruitment of memory T cells occurs prior to degenerative features of AMD in a murine mouse model. *Cx3cr1*-deficient mice manifest naturally occurring RPE degeneration and drusen formation in the course of aging. 8-month-old animals exhibit a pro-inflammatory state without apparent morphological changes in RPE. 12-month-old animals show a pro-inflammatory state with apparent RPE degeneration. We analyzed ocular samples of *Cx3cr1* (referred to as *Cx3cr1* in the following) at 8 and 12 months of age. While analyzing RPE-choroid complexes, we detected a significant increase in the number of T cells (CD3^+^) compared to *C57Bl/6J* control (**Figures 1C-E**). More specifically, T cells were largely detected at the vicinity of patches of RPE seemingly in transition to degeneration, not inside but in the close periphery of the degenerative area. (**Figure 1D**). T cells in the *Cx3cr1* retina displayed a wide morphological variance and consolidating activated phenotypes (**Figure 1F**). Furthermore, orthogonal sectioning and three-dimensional reconstruction of the confocal images confirmed that T cells were intimately associated to the basolateral membrane of RPE, suggesting a migration from the choroid and adherence to the RPE (**Figures 1G, H**).

Collectively, our findings support that T cells accumulate during early AMD prior to degeneration in our mouse model, and that these cellular populations are associated to the RPE.

### *Cx3cr1* mice display a specific ocular pro-inflammatory immunophenotype with high T cell frequencies prior to AMD-like degeneration

Next, we sought to investigate the diversity of T cell phenotypes in AMD by flow cytometry. We evaluated the neuroretina-RPE-choroid complex to capture organ-specific immune signature at 8 (basal time-point) and 12 months of age (prior to degeneration) in *Cx3cr1* mice and compared to *C57Bl/6J* control. For comparison with the systemic immune signature, we included the spleen as a systemic lymphoid organ. At 8 months of age, we did not observe differences in the ocular immune phenotypes of *Cx3cr1* mice at the T cell level compared to control (**Figures 1I, J**), nor systemically (**Figures 1K, L**). Interestingly, with age (12 months), the ocular profiles of *C57Bl/6J* mice showed a significant reduction of frequencies of T cells (CD3^+^) and T helper cells (CD3^+^ CD4^+^), whereas the frequencies of CD3^+^ increased significantly in *Cx3cr1* (**Figure 1J**). Importantly, these dynamics were only observable in the ocular samples, and the CD3^+^ and CD4^+^ T cell systemic populations were significantly decreased in both *C57Bl/6J* and *Cx3cr1* (**Figure 1L**). Furthermore, we found a significant increase of cytotoxic T cells (CD3^+^ CD8^+^) specifically in the eyes of *Cx3cr1* during aging (**Figure 1J**). Conversely, cytotoxic T cells remained stable in the spleen (**Figure 1L**). Together, these changes led to a significantly lower ratio of CD4^+^/CD8^+^ T cells in *C57Bl/6J* and *Cx3cr1* mice, suggesting a lymphocyte immunosenescence and active recruitment of cytotoxic T cells prior to the destructive features of retinal degeneration.

Complementarily, we evaluated potential sources of innate immune inflammation, yet no differences were found regarding myeloid cell frequencies (CD45^+^ CD11b^+^) at these time-points (**Supplementary Figures S3A–B**). We also evaluated populations of T cells potentially interacting with the innate response, but we could not observe changes in γδT cells (CD3^+^TCRγδ^+^) in the eye, albeit a decrease of these populations was observed in the spleen of control animals (**Supplementary Figures S3A–B**).

Taken together, our data show an influx of memory T cells, specifically cytotoxic T cells to the eye prior to the manifestation of retinal degeneration in human and mice.

### Experimental AMD reveals organo-specific activation of cytotoxic and helper T cells in the retina

**Figure 2:**
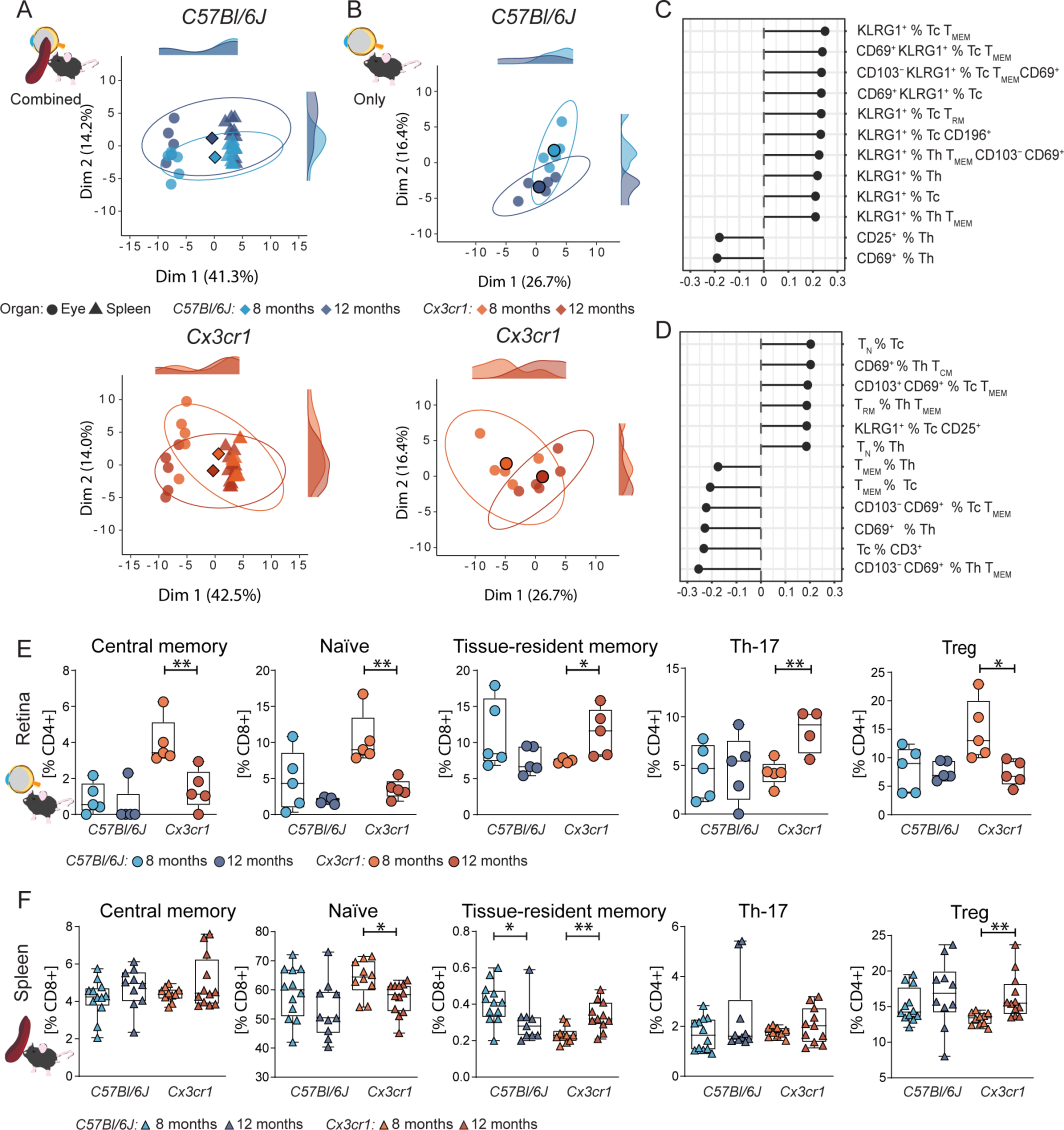
Retinal T cell subpopulations display age-related shifts in AMD. **(A-D)** Principal component analysis (PCA) using dimensional reduction on retinal and splenic immune cells on flow cytometric data. X and Y axis showing the first and second most contributing dimensions. Along the axis representative density plots are shown for the 8- and 12-month groups. Saturated symbol represents mean of a group. N= 5/group **(A)** PCA of systemic (spleen) and ocular immune data reveal a specific retinal immune population in both wild-type *C57Bl6/J*, (top), and AMD *Cx3cr1* mice (bottom). N= 10-12/group. **(B)** PCA of ocular immune populations captures an age-dependent shift in *C57Bl6/J* (top) captured by Dim2 and *Cx3cr1* (bottom) captured by Dim1 and partly by Dim2. **(C, D)** Loading plots for Dim1 and Dim2 illustrate the contribution of individual immune populations in *Cx3cr1*. Quantification of driver immune cells from the loading analysis originating from the eye **(E)** (N=4-5/group) and spleen **(F)** (N=10-12/group). Central memory T cells defined as CD44^+^ CD62L^+^, naïve as CD44^-^ CD62L^+^, tissue-resident memory as CD44^+^ CD62L^+^ CD69^+^ CD103^+^, Treg as CD25high and Th17 as CD196^+^. All comparisons with Student’s T-test test or Mann-Whitney U test. *p>0.05, ** p>0.01.

We used dimensionality reduction approaches to determine whether the T cell profiles in the context of experimental early AMD are organ specific. Using a principal component analysis (PCA) of hierarchically gated T cell subpopulations, we show a specific immunophenotype in the ocular samples (neuroretina-RPE-choroid) compared to the spleen in both *C57Bl/6J* and Cx3cr1 mice (**Figure 2A**). PCA analysis of the ocular populations revealed a strong time-point-dependent immunophenotype in the *Cx3cr1* mice compared to control (**Figure 2B**). To study the dynamics of T cell subpopulations, we analyzed PCA loadings. Our analysis indicates that changes in memory and activated T cell subsets are important for immune phenotype changes over time (**Figures 2C, D**). Age-related shifts in *Cx3cr1* mice were mainly driven by KLRG1^+^ and CD69^+^ subsets of memory T cells (Dim1, **Figure 2C**), known markers for effector functions in T cells as well as tissue residency, and participating in premature T cell immunosenescence.

### Early hit of specific retinal T memory populations and priming of resident retinal memory T cells

Since the PCA analysis revealed the potential contribution of memory T cell subsets, we subclustered T cells according to their CD44 and CD62L expression. At 8 months of age, *Cx3cr1* mice did not present manifestations of retinal degeneration, yet we observed a strong upregulation of central memory T cells (CD44^+^CD62L^+^) within their ocular immunophenotype compared to *C57Bl/6J* controls (**Figure 2E**). Importantly, these changes were exclusively found in the eye and not systemically (**Figure 2F**). In *C57Bl/6J* control mice, central memory T cells were hardly detectable. Similarly to the central memory T cells, we found an early upregulation of naïve T cells (CD44^-^CD62L^+^) at 8 months in *Cx3cr1* mice. Over time, central memory and naïve T cell frequencies decreased. On the other hand, the number of tissue-resident memory T cells (CD44^+^CD62L^−^CD103^+^CD69^+^) increased significantly, while this population tended to decrease in controls. These dynamics of naïve memory T cell regress and tissue-resident memory T cell upregulation was even detected systemically (**Figure 2F**).

Our T cell analysis suggests a role for an early hit of the memory T cell system in AMD pathogenesis before the occurrence of degenerative changes in the retina. Thus, we consider the adaptive immune system as one of the causative elements in the etiology of retinal degeneration in this model.

### Organo-specific dynamics of early Treg-like cells and late Th17-like cells

We sought to investigate regulatory T cells (Treg) and Th17 cells as prototypic anti- and pro-inflammatory T cells in central nervous system (CNS) inflammation, respectively. Recently studies reported alterations of these T cell subsets in AMD patients and animal models (16). In accordance with the literature, we observed a retina-specific increase of Th17-like cells (CD3^+^ CD4^+^ CD196^+^) in *Cx3cr1* mice, whereas no frequency alteration was detected in *C57Bl/6J* (**Figure 2E**). No trend was detected in systemic CD196^+^ Th17-like populations (**Figure 2F**).

Focusing on Treg (CD3^+^ CD4^+^ CD25^high^), we observed an upregulation at 8 months, paralleled by the upregulation of central memory and naïve memory T cells (**Figure 2E**) in *Cx3cr1* mice. Over time, ocular Treg decrease in *Cx3cr1*, whereas conversely a systemic Treg increase became apparent (**Figure 2F**). No dynamic in ocular nor systemic Treg population was detected in *C57Bl/6J*.

Overall, this pattern indicates an early anti-inflammatory response that precedes the disease phenotype in the retina. Importantly, this anti-inflammatory pattern was lost in the diseased retinal states.

### Ocular T cell populations express markers for activation and tissue residency throughout cytotoxic and helper T cells

Considering the changes in T cell surface markers KLRG1 and CD69 in the aging retina, we sought to characterize the activation status of cytotoxic and helper T cells. Specifically, within cytotoxic T cells, CD69 and KLRG1 known markers for cell activation and tissue residency were regulated in retinal immune populations.

**Figure 3:**
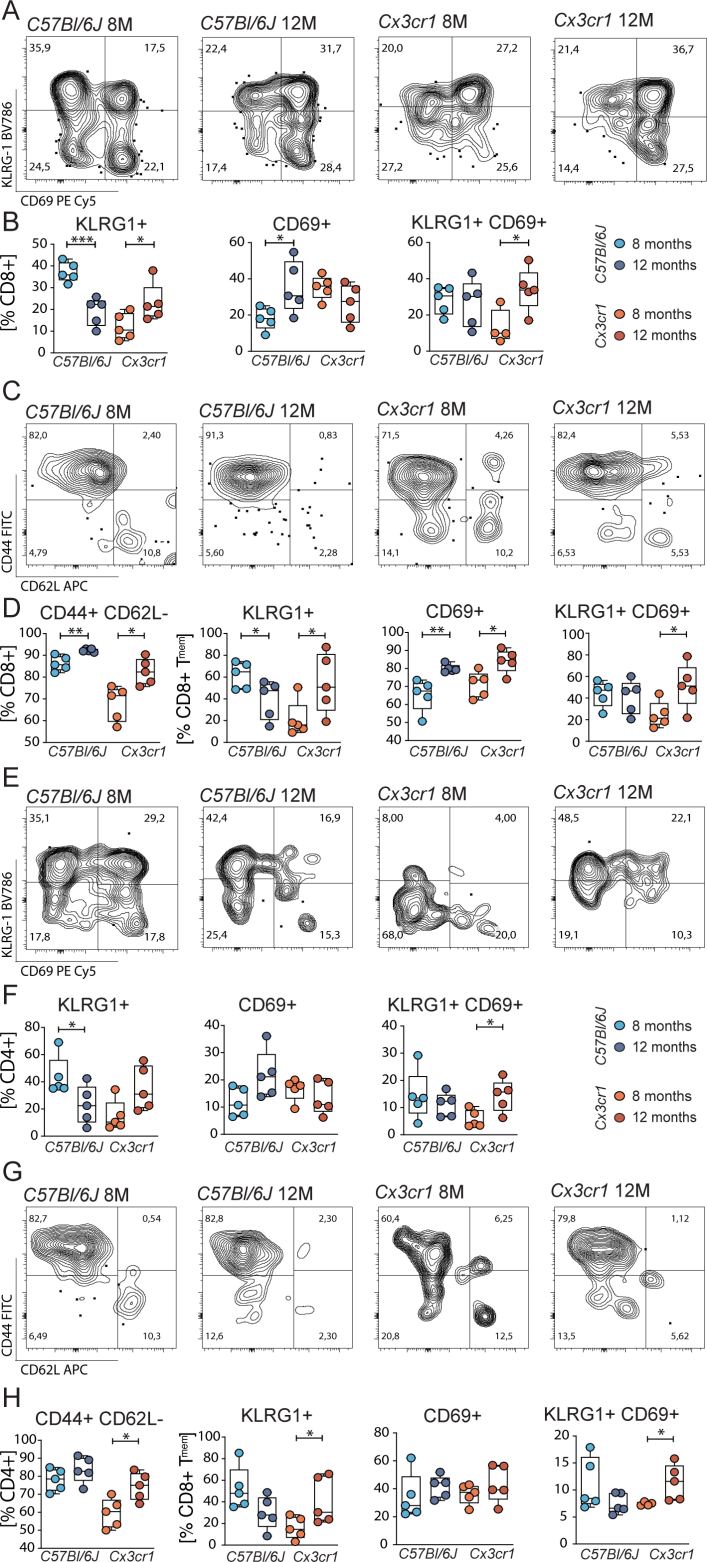
Altered KLRG1 and CD69 in retinal T cells. **(A)** Representative flow plots for CD69 and KLRG1 in ocular cytotoxic T cells from *C57Bl6/J* and *Cx3cr1* mice. **(B)** Quantification of KLRG1^+^, CD69^+^ and double positive cytotoxic T cells. N= 4-5/group **(C)** Representative flow plots for CD44 and CD62L in ocular cytotoxic T cells from *C57Bl6/J* and *Cx3cr* mice. **(D)** Quantification of effector memory (T_MEM_) cytotoxic T cells and KLRG1^+^, CD69^+^ and double positive cytotoxic T_MEM_ cells. N= 5/group. **(E-H)** Representative flow plots for CD69 and KLRG1 in ocular T helper cells from *C57Bl6/J* and *Cx3cr1* mice and their quantification. N= 5/group. All comparisons with Student’s T-test or Mann-Whitney U test. *p>0.05, ** p>0.01, ***p>0.001.

In retinal tissue of *Cx3cr1*, we observed an increase KLRG1^+^ as well as CD69^+^ KLRG1^+^ double positive cells over time. These changes were not found in *C57Bl/6J*; in contrast, we rather found a decrease of KLRG1 from the timepoint 8 months to 12 months (**Figures 3A, B**). In comparison, splenic CD8^+^ cells did not exhibit an alteration of neither KLRG1 nor CD69 (**Supplementary Figure S4B**), indicating a local retinal activation of the specific immune system in *Cx3cr1* mice. For T helper cells, we found a similar pattern of activation and antigen experience (**Figures 3E, F**). Systemically, we observed an upregulation of activated CD69^+^ T helper cells in *Cx3cr1* but no alteration in *C57Bl/6J* (**Supplementary Figure S4F**).

Since KLRG1 and CD69 are predominantly expressed in effector memory T cells, we focused our analysis on these subtypes. In *C57Bl/6J* and *Cx3cr1*, the number of cytotoxic effector memory T cells increased during aging (**Figures 3C, D**). This dynamic was specific for retinal populations, since the systemic analysis exhibited stable frequencies of these memory populations (**Supplementary Figure S4D**). Although both groups expressed the activation marker CD69 at 12 months to a greater extent, only *Cx3cr1* mice displayed an elevation of KLRG1 within the cytotoxic effector memory T cell population. In contrast, *C57Bl/6J* mice showed a decrease KLRG1 expression over time. Additionally, we found an increase in CD69^+^ KLRG1^+^ cells over time in *Cx3cr1* mice. These activation status effects were reflected in the systemic analysis of CD8^+^ effector memory T cells in *Cx3cr1* mice, whereas a downregulation of CD69^+^ KLRG1^+^ population in *C57Bl/6J* controls became apparent (**Supplementary Figure S4D**). Regarding T helper cells, no changes in the frequency or activation markers of effector memory cells were detected in *C57Bl/6J* (**Figures 3G, H**). However, the frequency of retinal CD4^+^ effector memory population in *Cx3cr1* increased over time. Retinal KLRG1^+^cells as well as CD69 KLRG1 co-expressing populations within the CD4^+^ effector memory T cells were dynamically upregulated from timepoint 8 to 12 months. Systemically, we observed an increase in the number of CD4^+^ effector memory T cells in *Cx3cr1* mice. Additionally, a systemic upregulation of KLRG1 and CD69 can be observed in the CD4^+^ effector memory compartment of the *Cx3cr1* mice. (**Supplementary Figure S4H**) This again contrasts with the unaltered systemic effector memory T cells of *C57Bl/6J*, corroborating a systemic impact of retinal activated T cells in the *Cx3cr1* animals.

In summary, our flow cytometric analysis reveals upregulation of markers for tissue residency and effector function of T cells specifically in the retina/RPE/choroid complex of *Cx3cr1* mice.

### Aging of *Cx3cr1* mice shifts pro-inflammatory gene expression of the RPE-choroid complex and supports the presence of local micro-inflammation

Immunophenotyping of T cell populations suggests the recurrence of interactions among different cells, including memory T cells and other pro- and anti-inflammatory subpopulations. To provide further evidence for this active immunomodulatory scenario specific to the retina and choroid, we profiled gene expression of relevant genes involved in inflammation in neuroretina and in RPE-choroid complex samples.

**Figure 4:**
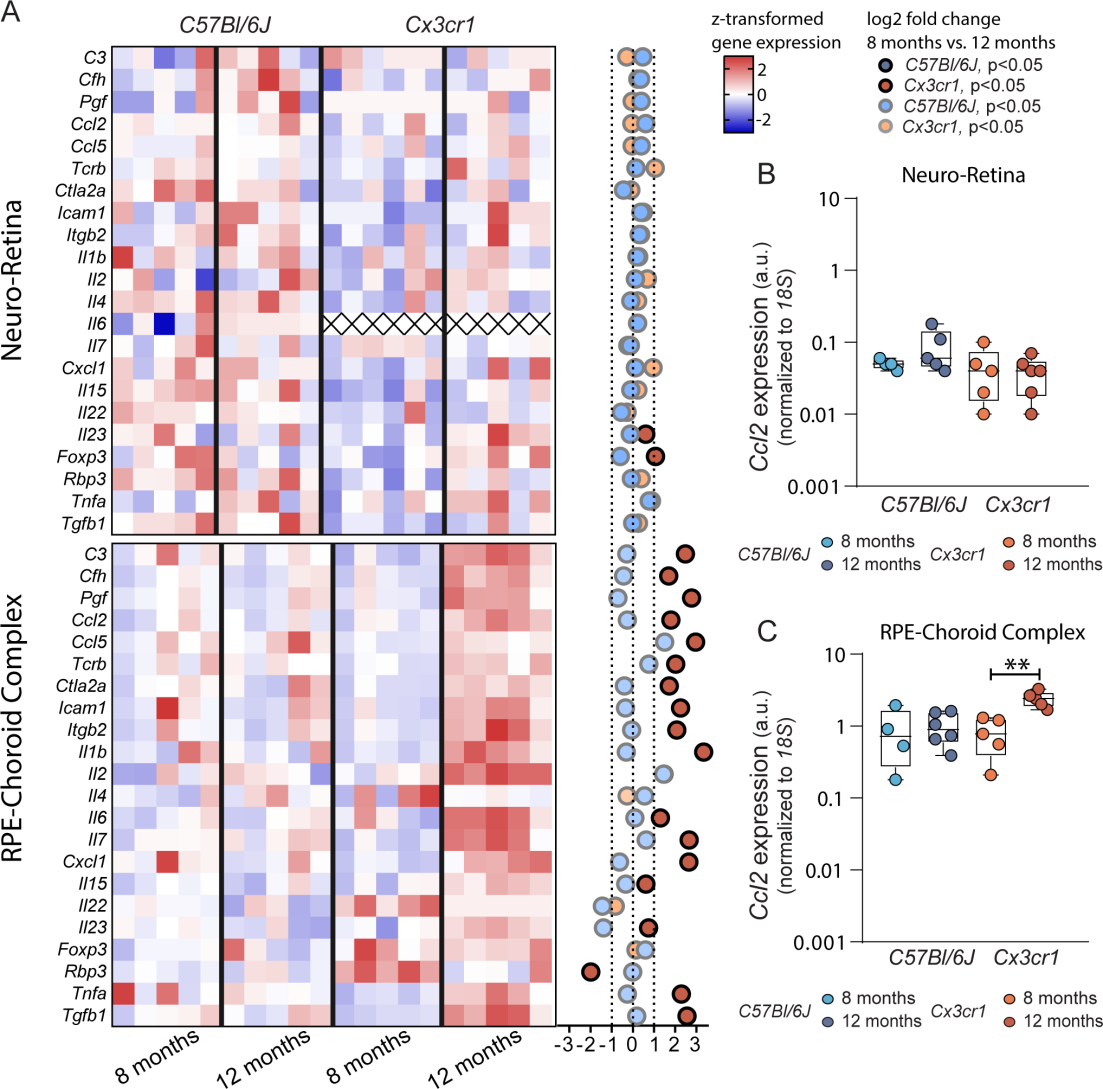
Altered ocular gene expression in age-related macular degeneration. **(A)** Heatmap of z-scored gene expression for several inflammation and AMD-related genes in the Neuro-Retina (top) and RPE-Choroid-Complex (bottom). Dot plot to the right indicates overall log2FC between 8 months and 12 months (N= 5-6/group). Alpha indicates significance. All comparisons with Student’s T-test. Gene expression for Ccl2 shown as boxplot for Neuro-Retina **(B)** and RPE-Choroid-Complex **(C)**. (N= 4-6/group). Comparisons with Student’s T-test. ** p>0.01.

Globally, the inflammatory signature found in the neuroretina was modest, showing only an upregulation of transcription factors *Il23* and *Foxp3* in 12-month-old *Cx3cr1* mice (**Figure 4A**). In the RPE-choroid complexes, where most observed degenerative features take place, we observed a transcriptomic activity ∽10-fold higher to baseline (**Figure 4B**). Furthermore, we observed an age-dependent dynamic shift (8 months to 12 months of age) only in *Cx3cr1* mice, and not in *C57Bl/6J* control. The most expressed interleukins found in aged *Cx3cr1* RPE-choroid samples *were Il1b, Il2, Il6, Il7, Il8 (*CXCL1*), Il15 and Il23*. *Tcrb* was strongly upregulated along with genes coding for the adhesion and activation molecules *Icam1* and *Itgb2* (CD18). Moreover, aging also influenced the expression of genes known to contribute to AMD pathogenesis, including *C3*, *Cfh*, *Pgf*, *Tnf*, *Tgfb* and *CCL2* (1, 5). Other genes such as *Ctla2a* were also increased over time in the *Cx3cr1* RPE-choroid, whereas the expression of *Rbp3* was significantly decreased.

## Discussion

The presence of lymphocytes and retinal autoantibodies in AMD was firstly discussed in 1984 by Penfold and colleagues (24–26). Two more decades were necessary to validate these data histologically in patients with GA (9). Yet, the specific mechanisms and contribution of the adaptive immune system to AMD have remained elusive. Here, we provide for the first time a comprehensive and comparative analysis of T cell frequencies in the context of AMD progression using mouse models and validation in human samples. Importantly, we demonstrate that the recruitment of memory T cells occurs at early stages of AMD and prior to the degenerative hallmarks of AMD and GA. We also show that retinal/choroidal cellular immune phenotype is distinct from the systemic phenotype. While the numbers of regulatory T cells decrease with age, CD196^+^ Th17-like cell populations accumulate in the diseased retina and become effectors of degeneration. Altogether, this work underscores a novel view on the pathophysiology of AMD and sheds light onto potential new therapeutic targets aiming at adaptive immunity players.

Under physiological circumstances, the RPE wields a strong immunomodulatory ability to preserve an active barrier *via* multiple soluble and membrane bound factors (27). Among its functions, the RPE can suppress T cell activation and skew T cell populations towards a regulatory phenotype (28–30). The control and interplay of the RPE with the adaptive immune response following damage is, however, not fully understood. In conditions such as aging or AMD, our group has previously reported that FoxP3 expression can alter the immunomodulatory functions of the RPE and affect its ability to govern T cell populations (31). Herein, our data illustrates how the transmigration and accumulation of T cells in the retina during early AMD is associated to regions of the retina transitioning towards RPE degenerative profiles. Specifically, T cells with a memory phenotype exhibit highly activated cytotoxic features which contribute to long lasting neuroinflammation (32–34). Phenotypic dendritic shaped T cells are strongly suggestive of patrolling local tissue-resident memory T cells (35–38), Additionally, the T cell morphology that includes a bloated cell diameter, endocytic or diapedetic characteristics provides fundamental evidence of the activated state of the adaptive immune system (39–41). We observed the emergence of T cells in the diseased retina prior to the significant RPE injuries, thus indicating that the adaptive immune response is likely to take place at early stages of AMD when the clinical manifestations are not evident yet. Cellular stress from aging as well as chronic inflammation are known to render the immunogenic properties of the RPE more permissive by altering their secretory profile other plasma membrane receptors (42). Our findings confirm that T cells invading ocular tissues express high levels of molecules promoting adherence and transmigration such as *Icam1* and *Itgb2* (43, 44). We also observed enrichment in cytokines participating in T cell migration such as *Ccl5* (45) and other immunomodulatory factors like *Foxp3* and *Ctla2a* (31, 46). In fact, T cells may bypass the immune regulatory RPE monolayer upon antigen activation independent of these classical adhesion and migration signals by inducing apoptosis in RPE cells (47) and altering the interactions of the RPE with the immune system (27, 48). Given that CX3CR1 is a key regulator of the intercommunication of myeloid cells and the adaptive immune system in the CNS (49), it remains the question whether CX3CR1^+^ T cells have a higher migratory ability in other models as well as in AMD patients. This, however, is unlikely, and others have reported that cytotoxic T cells from blood of patients with neovascular AMD display decreased frequencies of CX3CR1^+^ T cell populations, whereas CX3CL1 plasma levels remained unaltered compared to control (50, 51). Considering the transgene background of our experimental AMD model, we conclude that T cell adherence to the RPE and transmigration relies on signaling alternative to the CX3CR1 migration pathway (52).

Albeit studying the adaptive immune system in the retina is challenging due to the spatial and temporal dynamics of the immune response, a number of studies attempted this approach in peripheral blood of patients with AMD. To a certain extent, analyses of human peripheral T cell frequencies provided evidence for the significance of primed T cells in the pathology of AMD. One study showed that in a 30-day period of the diagnosis neovascular AMD, peripheral lymphocyte frequencies significantly raised compared to a cohort of patients who received their diagnosis longer than 30 days before or after blood sampling (53). Additionally, studies using a mixed cohort of dry and neovascular AMD patients revealed that peripheral cytotoxic T cells are enriched in CD56. Interestingly, the presence of CD56^+^ T cells associated with an increased 3.2-fold risk of developing AMD, which in patients harboring the AMD risk allele in the *CFH* gene (the Y402H polymorphism in the FH protein) translated to a 13.3-fold risk (54). Nevertheless, there is sufficient evidence supporting the notion that cell-based inflammatory responses and core features to AMD can directly originate in the RPE and choroid, even, at earliest potential stages of AMD (55). Because of that, our observations with regard the shifts in tissue-specific T cell populations in the degenerating AMD-like retina showcase a strong correlation to aging and suggest the intrinsic dependency to gradual accumulation of damage by the RPE. More particularly, the increase of CD69^+^KLRG1^+^ T cell populations in the diseased retina underlines the immune activation inherent to the tissue and involving key adaptive immune players. Whereas KLRG1^+^ T cells are typically short-living effector cytotoxic T cells with antigen experience (56–60), the expression of KLRG1^+^ in T helper cells may mark a proliferative history rather than an activation state (61). Nevertheless, KLRG1^+^ T cells show a high capacity to transform into functional memory T cells (62, 63). Moreover, this increase of frequencies of CD69^+^KLRG1^+^ cells in the murine model (of note: there was no difference in the CD4 subpopulations between WT and *Cx1cr3* knockout model excluding a model bias) and CD45RO^+^ cells in the human choroid also reflect a turnover of the T memory compartment. The expression of these markers in effector memory T cells points at the regulation of effector genes and transcription factors with long-lasting properties (56). Additionally, KLRG1 expression facilitates formation of tissue-resident memory cells (64). Correlating with that, we found an enrichment of IL-2, IL-7 and IL-15 as pivotal cytokines for development, homeostasis and maintenance of memory T cells (65–68). Altogether, these populations usher in processes of destruction of the RPE integrity and the genesis of long-lasting T-cells that will, ultimately, promote low-grade chronic inflammation through the innate immune response.

Besides the striking activity that we have observed in the memory T cell compartment in experimental retinal degeneration, we also regarded CD196^+^ Th17-like and regulatory T cells as potential disease-contributing cell populations in our model and in AMD. In fact, we found that while the frequencies of CD196^+^ Th17-like cells increased by time, the Treg cell frequencies decreased in the degenerating retina. Others have suggested that a decrease in Treg cell populations could be explained by the immunomodulatory defects in the RPE, which can lead to convert helper T cells to Treg cells in the presence of CTLA2A. This mechanism, however, seems to fail with time in retinal inflammation (30, 69). Additional *in vivo* models of chronic inflammation in T cell-mediated uveitis showed that a pro-inflammatory environment can dampen Treg functionality (70) and lead to a reduction of Treg cell frequencies (71, 72). However, comparing systemic Treg frequencies in patients with neovascular AMD to healthy controls, Madelung and colleagues did not find a significant difference (73). Conversely, *Cx3cr1* mice showed a systemic but not tissue-specific increase of Treg frequencies. These features support our hypothesis that the observed shifts of T cell profiles in the RPE and choroid are a product of the ongoing retinal degeneration and no due to lack of expression of Cx3cr1. Future studies are needed to further understand the balance of T helper cell subtypes in AMD, including Th1, Th2, and others, including the staining of specific transcription factors. While CD25 captures Treg nicely, CD196 only indicates a Th17-like population enriched for RORγt expression. Given our limitation to access to additional human specimens, we were not able to screen for CD44, CD69 and KLRG1 cell populations in ocular tissues as observed in our murine model. Additional translational studies should address this avenue.

Collectively, this work provides a comprehensive insight to the dynamics of cell populations of the adaptive immune system at both the retina and systemically in a model of early AMD. Our data corroborates preexisting literature findings and recognizes T cells, especially of the T memory compartment, as potential drivers of AMD at early stages. Altogether, our study leads to the presumption of an *“early hit”* model by the adaptive immune system during AMD. For the first time, we showed the presence of CD69^+^KLRG1^+^ cells prior to RPE degeneration in an experimental GA mouse model as well as CD3^+^CD45RO^+^ cells in human retinal specimens. The presence of these populations of cells at early stages of AMD proves the participation of the learning T cell-depending immune system. Together with the recent notions on complement factors (74, 75), the immunogenic associations of the adaptive immune system in AMD foster the potential to become new therapeutic targets in the landscape of immune players participating to retinal degeneration.

## Data Availability

The original contributions presented in the study are included in the article/**Supplementary Material**. Further inquiries can be directed to the corresponding author.
