# Supplementary material for: Outer retina micro-inflammation is driven by T cell responses prior to retinal degeneration in early age-related macular degeneration
Source: Front Immunol. 2025 Feb 5;16:1520188. doi: 10.3389/fimmu.2025.1520188 (PMC11835843; doi:10.3389/fimmu.2025.1520188)
Supplement: Supplementary file 1 [file DataSheet1.pdf]

SUPPLEMENTARTY MATERIAL

Outer retina micro-inflammation is driven by T cell responses prior to retinal degeneration in early age-related macular degeneration

Authors: Lucas Stürzbecher[1,2,3,4], Hendrik Bartolomaeus[2,3,5,6], Theda U. P. Bartolomaeus[2,3,5,8], Sylvia Bolz[9], Andjela Sekulic[1], Marius Ueffing[9,10], Simon J. Clark[9,10,11], Nadine Reichhart[1], Sergio Crespo-Garcia[7], Nicola Wilck[2,3,5,12], Olaf Strauß[1,*]

Affiliations:

[1]Experimental Ophthalmology, Department of Ophthalmology, Charité - Universitätsmedizin Berlin, Corporate Member of Freie Universität and Humboldt Universität zu Berlin, Berlin, Germany.

[2]Experimental and Clinical Research Center, a cooperation of Charité-Universitätsmedizin Berlin and Max-Delbrück-Center for Molecular Medicine, Berlin, Germany.

[3]Max-Delbrück-Center for Molecular Medicine in the Helmholtz Association, Berlin, Germany.

[4]Eye Center, Medical Center, Faculty of Medicine, University Medical Center Freiburg, Freiburg, Germany.

[5]DZHK (German Centre for Cardiovascular Research), partner site Berlin, Berlin, Germany.

[6]Institute of Experimental Biomedicine, University Hospital Würzburg, Würzburg, Germany.

[7]École d'optométrie, University of Montreal, Montréal, Canada.

[8]Charité-Universitätsmedizin Berlin, corporate member of Freie Universität Berlin and Humboldt-Universität zu Berlin, Berlin, Germany.

[9]Institute for Ophthalmic Research, Department for Ophthalmology, Eberhard Karls University of Tübingen, Tübingen, Germany.

[10]Department for Ophthalmology, University Eye Clinic, Eberhard Karls University of Tübingen, Tübingen, Germany.

[11]Lydia Becker Institute of Immunology and Inflammation, Faculty of Biology, Medicine, and Health, University of Manchester, Manchester, UK.

[12]Charité - Universitätsmedizin Berlin, corporate member of Freie Universität Berlin and Humboldt-Universität zu Berlin, Department of Nephrology and Internal Intensive Care Medicine, Berlin, Germany.

***Correspondence to**: Olaf Strauß, Experimental Ophthalmology, Charité Universitätsmedizin Berlin, Campus Virchow, Augustenburger Platz 1, 13353 Berlin, Germany, phone: +49 30 450 654 359, e-mail: olaf.strauss@charite.de

List of supplementary material

Supplementary detailed methods

Histology of human AMD samples.

Histological evaluation of murine RPE-choroid complex whole-mounts.

Immune cell phenotyping using multi-color flow cytometry.

Quantitative real-time PCR of ocular and splenic tissues.

Supplementary figures

Supplementary Figure S1. Gating strategy for splenic immune cells.

Supplementary Figure S2. Gating strategy for retinal immune cells.

Supplementary Figure S3. Retinal and splenic immune populations.

Supplementary Figure S4. Systemic activation von $CD8^+$ and $CD4^+$ T cells and effector memory T cells.

Supplementary Figure S5. Principal component analysis of splenic immune cells stratified by timepoint.

Supplementary Figure S6. No relevant shift due to autofluorescence in *Cx3cr1* animals in FITC channel.

Supplementary tables

Supplementary Table S1. List of human specimens.

Supplementary Table S2. Flowcytometric Reagents.

Supplementary Table S3. Histological Reagents.

Supplementary Table S4. Genes and Primers for qPCR.

Supplementary detailed methods

Histology of human AMD samples.

The sagittal sections were thawed at room temperature, permeabilized with 0.3% Triton X-100 in PBS and treated with the True Black Lipofuscin Autofluorescence quencher reagent (Linaris, 23007) to reduce autofluorescence. Tissue was incubated with primary antibodies CD3 (Roche/Ventana, 790-4341, 0,4µg/ml) and CD45R0 (DAKO, M0742, 1:100) for 16 hours at 4°C. After washing, tissues were incubated with secondary antibodies for 1 hour at room temperature and counterstained with DAPI (Thermo Scientific, 62248). Images were obtained using a Leica TCS SPE confocal microscope (Leica Microsystems GmbH, Wetzlar, Germany). $CD3^{+}CD45R0^{+}$ cells were counted per high power fields (HPF) at a magnification of 40x and analyzed using Leica Application Suite X Software and *ImageJ* (https://imagej.net/ij/). A detailed list of the antibodies used in this study is available in **Supplementary Table S2**.

Histological evaluation of murine RPE-choroid complex whole-mounts.

After enucleation and dissection of fat, muscles and connective tissue around the bulbus, eye globes were kept in 4% paraformaldehyde for 12 minutes. For whole-mount preparation, a circumferential incision behind the limbus was performed, and the cornea, lens and ciliary body were discarded. Four to five radial incisions eased to flatten the whole-mount. The neuro-retina was lifted via cutting the opticus, and the remaining RPE-choroid complex was kept for histological evaluation. For ideal antibody penetration, tissue was permeabilized in TBS with 5% Triton X-100 for 24 hours at 4°C. After washing three times in TBS for 10 minutes, samples were subjected to blocking buffer (TBS with 5% BSA) for 60 minutes. Different primary antibodies were incubated for 48 hours at 4°C. RPE-choroid complexes were stained with anti-CD3 (1:100) and Acti-stain^{TM} Phalloidin (1:250), and signal was retrieved with secondary antibodies anti-rabbit (1:250) in DAPI working solution (300nM) for 60 minutes at room temperature. A detailed list of antibodies is provided in **Supplementary Table S2**. Tissues were whole-mounted with Fluorescence Mounting Medium (Dako) and imaged using a Leica TCS SP8 DLS confocal microscope (Leica Microsystems GmbH, Wetzlar, Germany). Further image analyses were performed using Leica Application Suit X Software and *ImageJ* (https://imagej.net/ij/).

Immune cell phenotyping using multi-color flow cytometry.

Following enucleation and dissection of fat, muscles, connective tissue, remaining N. opticus, cornea, lens and ciliary body the neuroretina-RPE-choroid-complex was digested for 30 min in a PBS TL Liberase solution at 37°C under constant movement and tissue was later mechanically homogenized with a gentle pipetting. The cellular suspension was then filtered through a 40µm-mesh strainer (Greiner Bio-one). To increase the yield of cells, each probe consisted of a pool of 3 eyes (2 animals).

For spleens, single cell suspensions of the organs were obtained through filtering through 70µm- and 40µm-mesh strainers (Greiner Bio-one) applying mechanical pressure. Splenic suspensions were cleared through hypotonic erythrocyte lysis (155 mM NH4Cl, 10 mM NaHCO3, and 1 mM NaEDTA in water). Trypan blue was used to detect the yields of dead cells while counting. Each spleen (1 animal) was analyzed individually.

A probe sample of 10^6 cells were used for multicolor flow cytometric analyses. All flow cytometric measurements included dead cell exclusion using Live/Dead Fixable Aqua Dead Cell Stain Kit. Cells were stained with surface antibodies (**Supplementary Table S3**) in PBS + 0.5 %BSA + 2 mM EDTA together with F_c blocking reagent (Miltenyi) for 30 min on ice. Data were recorded with a BD FACS LSR Fortessa and processed with the BD FACS Diva Software. Analyses were performed using FlowJo™ Version 10.8.1 (BD). For immune cell analysis, Log transformation was used to normalize immune cell (features) counts. The gating strategies were set using Fluorescence Minus Ones Controls (FMOs) and are provided in **Supplementary Figure S1** and **Supplementary Figure S2**.

Quantitative real-time PCR of ocular and splenic tissues.

We determined gene expression in either RPE-choroid complexes or spleen. Upon excision, tissues were immediately snap frozen in liquid nitrogen and stored at -80°C. Total RNA was extracted with the RNeasy Mini Kit (QIAGEN) following the manufacturer's protocol, and concentration and quality measured with a NanoDrop-1000 Spectrophotometer (PeqLab). cDNA was synthesized from 300ng of RNA with the High-Capacity cDNA Reverse Transcription Kit (Applied Biosystems™, Thermo Fisher). The expression of target genes was detected with either TaqMan or SYBR Green systems using the standard curve method on a QuantStudio™ 3 software (Applied Biosystems™, Thermo Fisher). The expression of target mRNA was normalized to the expression of the *18S* housekeeping gene. TaqMan primers were purchased whereas SYBR Green primers were designed in PrimerExpress 3.0 (Applied Biosystems) and synthetized by Biotez (Berlin, Germany). All primers, probes and sequences are provided in the **Supplementary Table S4**.

Supplementary figures

Supplementary Figure S1 Gating strategy for splenic immune cells

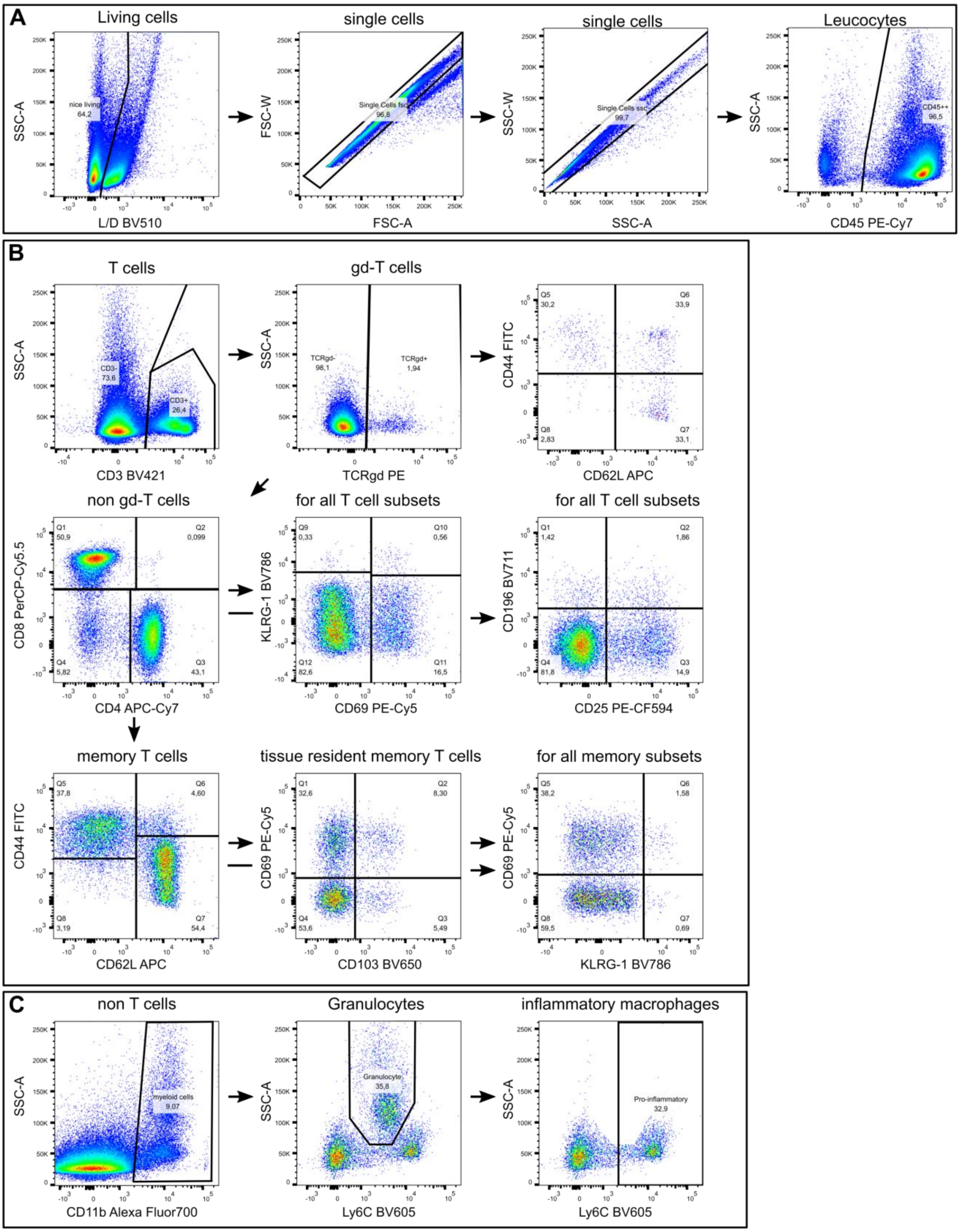

Figure S1. (A) Showing general gating strategy for splenic immune cells. **(B)** Gating strategy for T cells from which Treg-like ($CD25^+$), Th17-like ($CD196^+$), $CD69^+$ and $KLRG1^+$ cells were determined, as well as T memory cell populations. **(C)** for non T cells.

Supplementary Figure S2 Gating strategy for retinal immune cells

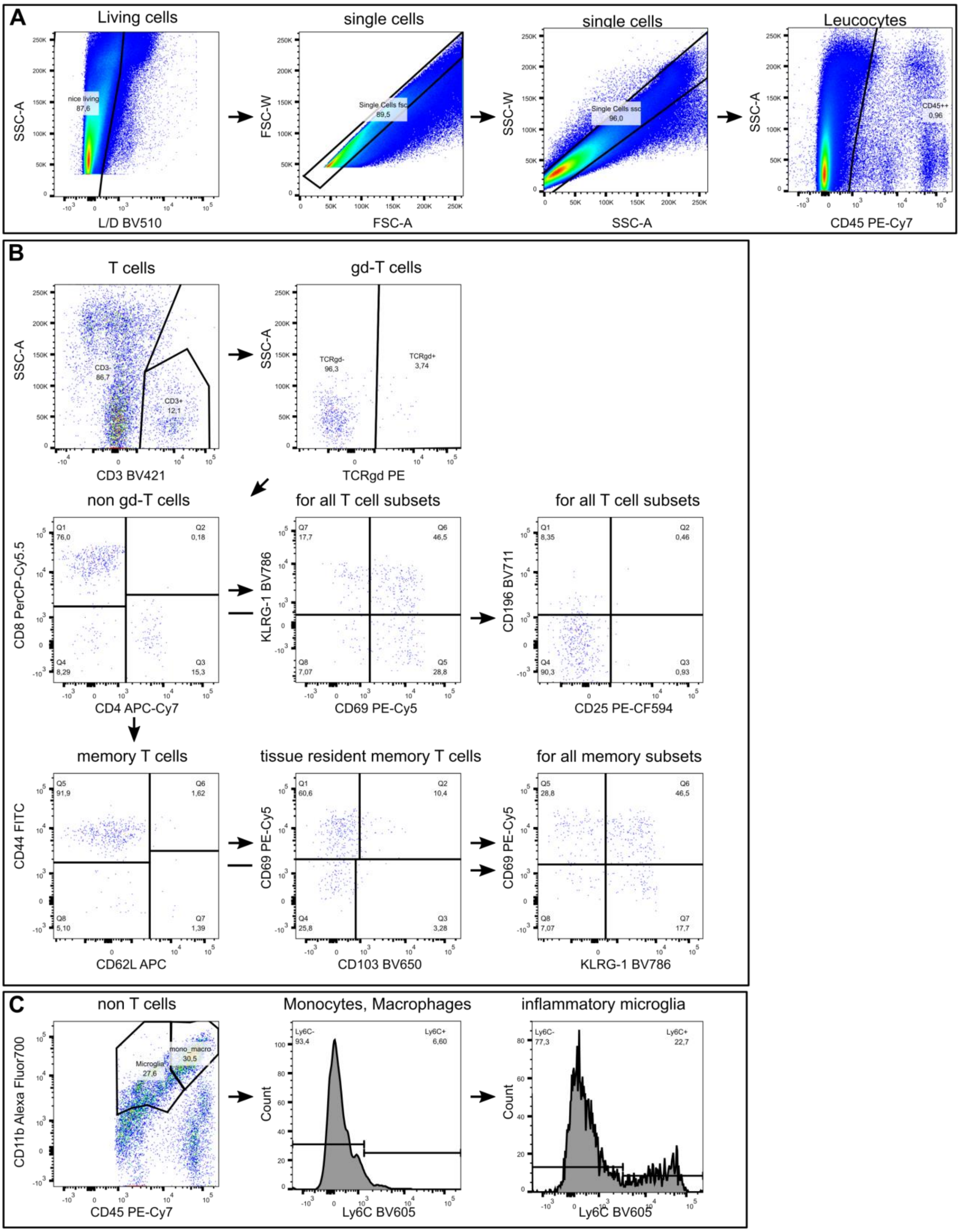

Figure S2. (A) Showing general gating strategy for retinal immune cells. **(B)** Gating strategy for T cells from which Treg-like ($CD25^+$), Th17-like ($CD196^+$), $CD69^+$ and $KLRG1^+$ cells were determined, as well as T memory cell populations. **(C)** for non T cells, inflammatory microglia and monocytes/macrophages respectively.

Supplementary Figure S3 Retinal and spenic immune populations

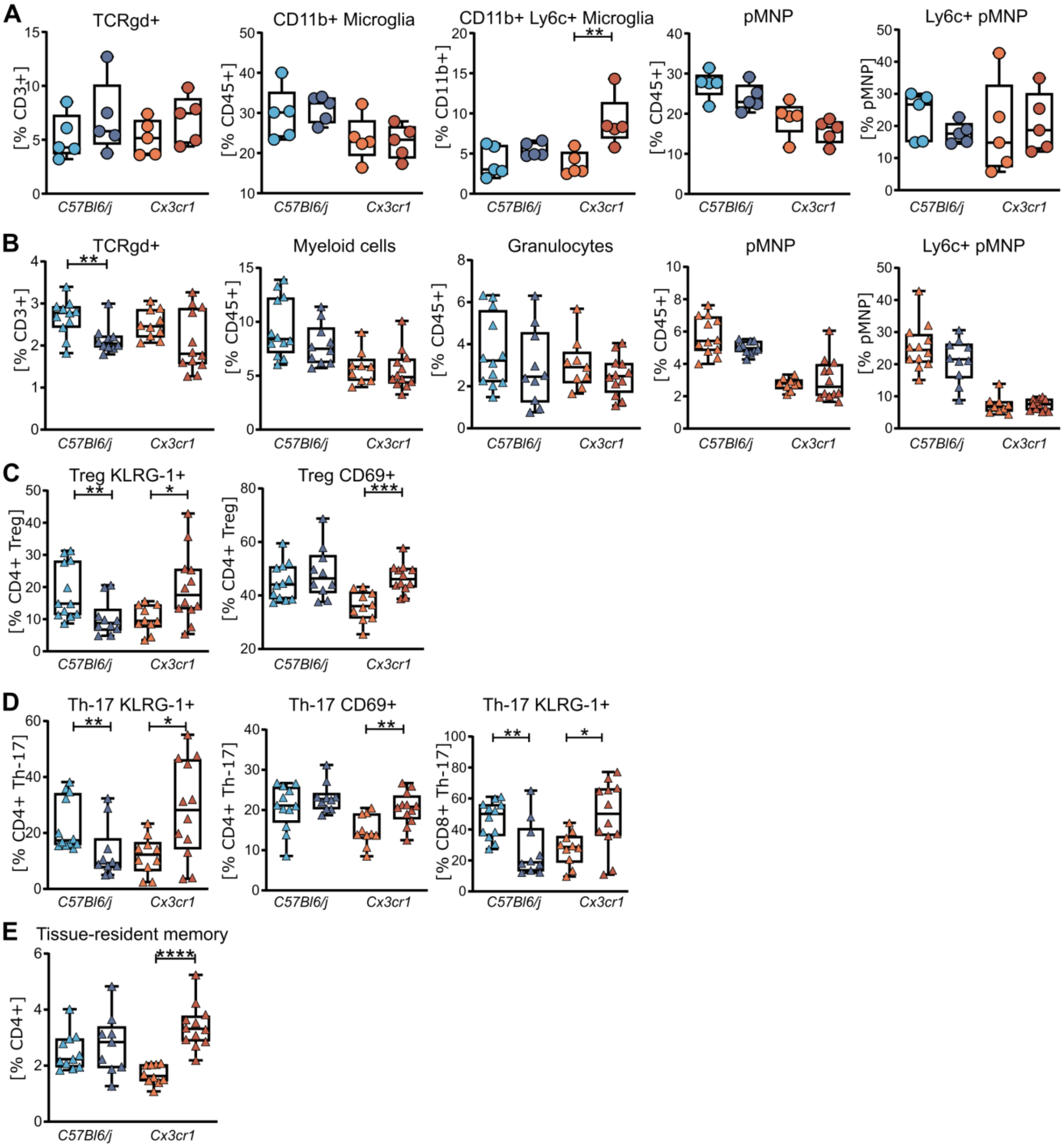

Figure S3. **(A)** Retinal TCRgd and non T cell immune populations. **(B)** Splenic TCRgd and non T cell immune populations. **(C)** KLRG-1 and CD69 marker expression on splenic CD4^{+} regulatory T cells. **(D)** KLRG-1 and CD69 marker expression on splenic CD4^{+} and CD8^{+} Th17 like cells. **(E)** Splenic CD4^{+} tissue-resident memory T cells. Tested with double sided t-test or Mann-Whitney U test. *$P < 0.05$; **$P < 0.01$; ***$P < 0.001$; ****$P < 0.0001$.

Supplementary Figure S4 Systemic activation of $CD8^+$ and $CD4^+$ T cells and effector memory T cells

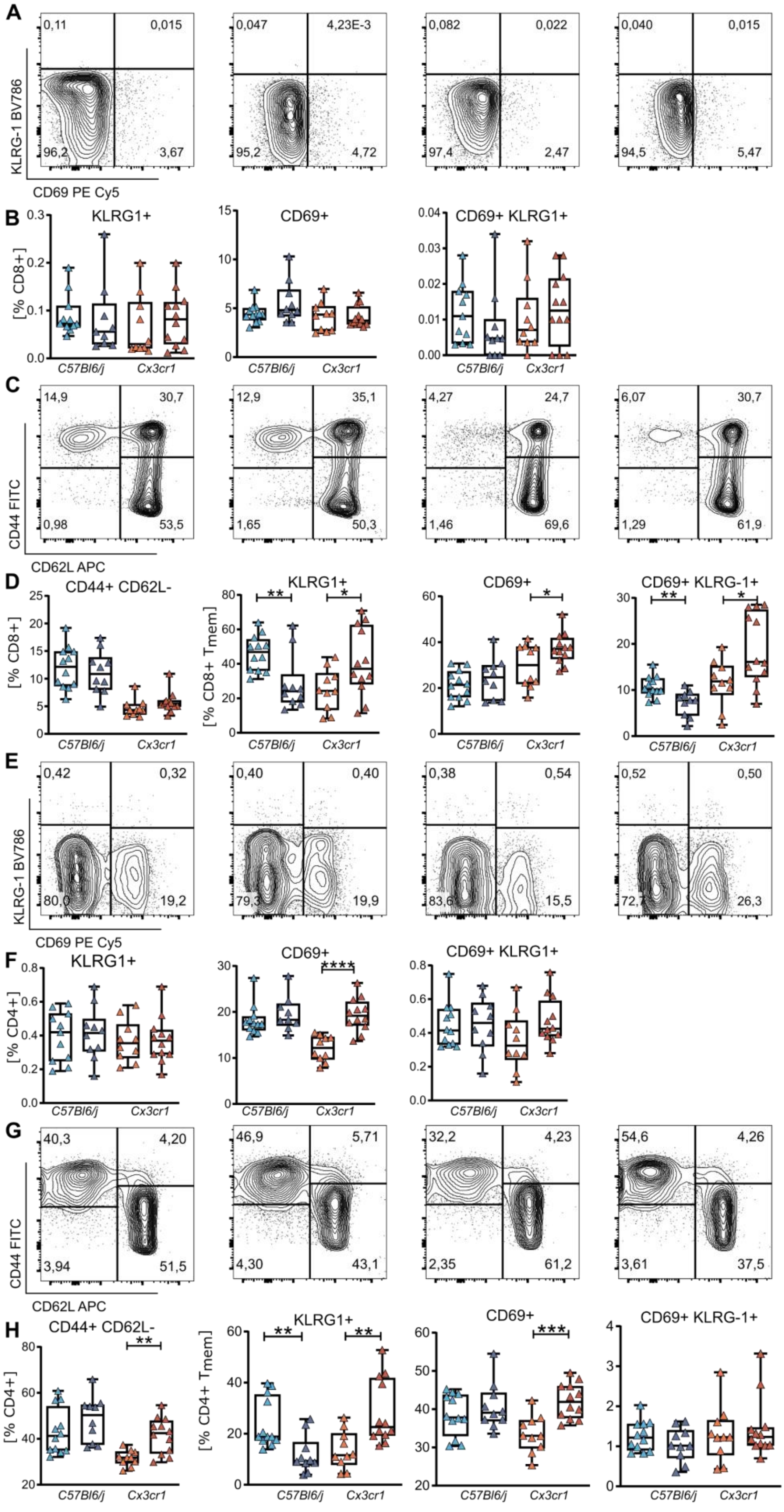

Figure S4. (A, C) Representative gates for KLRG1 and CD69 and CD44 and CD62L on splenic $CD8^+$ T cells. Numbers representing percentage of gated immune cells. **(E, G)** Same for splenic $CD4^+$ T cells. **(B, D, F, H)** Quantification of marker expression in **(B)** $CD8^+$ T cell population **(D)** $CD8^+$ effector memory T cell population ($CD44^+$ $CD62L^-$) **(F)** $CD4^+$ T cell population and **(H)** $CD4^+$ effector memory T cell population ($CD44^+$ $CD62L^-$). Tested with double sided t-test or Mann-Whitney U test. *$P < 0.05$; **$P < 0.01$; ***$P < 0.001$; ****$P < 0.0001$. 8M WT and 12M KO n = 12; 12M WT and 8M KO n = 10.

Supplementary Figure S5 Principal component analysis of splenic immune cells stratified by timepoint

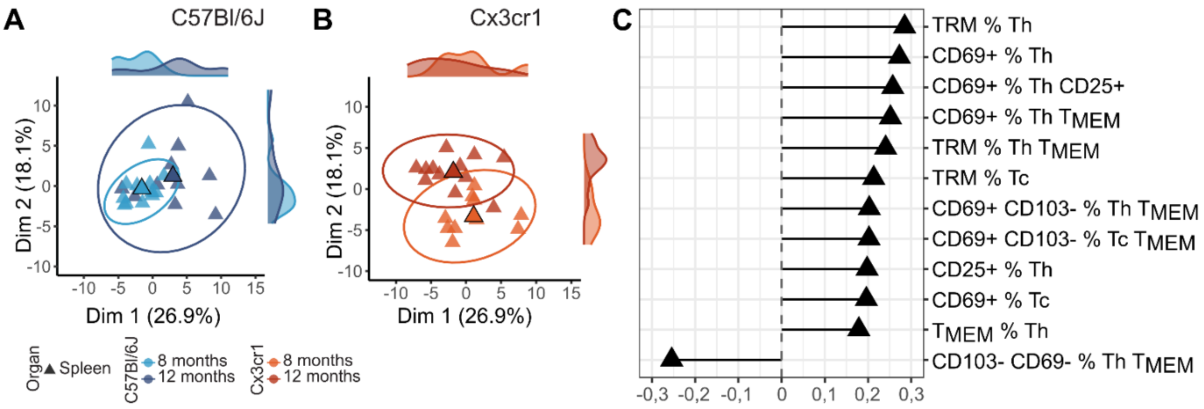

Figure S5. (A, B) Principal component analysis (PCA) of splenic immune cells. Each triangle represents a single animal. Saturated symbol represents mean of a group. **(A)** shows *C57bl6/j* **(B)** *Cx3cr1* animals, respective coloring indicates timepoint. **(C)** shows the 12 most contributing immune cell populations to dim2.

Supplementary Figure S6 No relevant shift due to autofluorescence in Cx3cr1 mice in FITC chanel

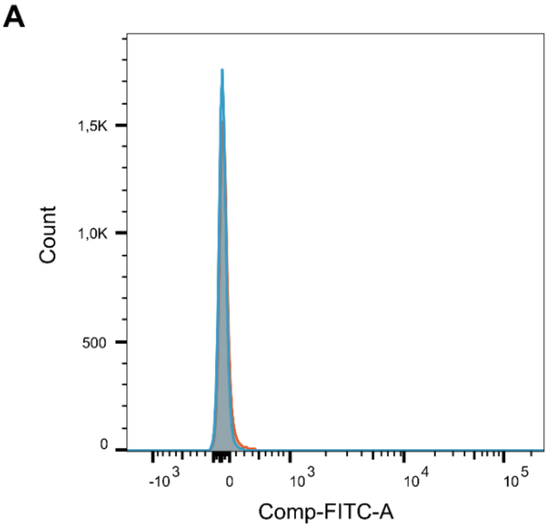

	Sample	Background	Count
	12 month wild type, spleen	*C57bl6/j*	10000
	12 month knockout,spleen	*Cx3cr1*$^{gfp/gfp}$	10000

Figure S6. (A) Showing FITC signal of unstained splenic cells from *C57bl6/j* (light blue) and *Cx3cr1* (orange) mice. Histogramm showing no relevant shift of green autofluerencence in FITC chanel from *Cx3cr1*$^{gfp/gfp}$ knockout.

Supplementary Tables

Supplementary Table S1. List of human specimens

Control			Early AMD			Late AMD		
ID	*Sex*	*Age (years)*	*ID*	*Sex*	*Age (years)*	*ID*	*Sex*	*Age (years)*
ETR104	M	68	ETR216	M	79	ETR64	M	69
ETR65	M	79	ETR631	F	71	ETR405	M	80
ETR314	F	71				ETR292	F	72
Average		72.6 ± 5.68	Average		72.6 ± 5.68	Average		73.6 ± 5.68

AMD: Age-related macular degeneration; ETR: Eye tissue resource; M: male; F: female

Supplementary Table S2. Flowcytometric Reagents

Antibody	Fluorophore	Clone	Identifier	Source	Concentration
Fc Blocking			130-092-575	Milteny Biotech	1:100
CD3 –	BV421	17A2	564008	BD Horizon	1:50
CD4 –	APC Vio770	GK1.5	130-118-957	Milteny Biotech	1:100
CD8 –	PerCP Cy5.5	53-6.7	551162	BD Pharmingen	1:100
CD11b –	Alexa Fluor 700	M1/70	557960	BD Pharmingen	1:100
CD25 –	PE CF594	PC61	562694	BD Horizon	1:100
CD44 –	FITC	IM7	553133	BD Pharmingen	1:100
CD45 –	PE Vio770	REA737	130-110-661	Miltenyi Biotech	1:100
CD 62L –	APC	MEL-14	553152	BD Pharmingen	1:100
CD69 –	PE Cy5	H1.2F3	104510	BioLegend	1:100
CD103 –	BV650	2E7	748256	BD OptiBuild	1:50
CD196 –	BV711	11A9	563923	BD Horizon	1:100
KLRG1 –	BV786	2F1	565477	BD Horizon	1:100
Ly6C –	BV605	AL21	563011	BD Horizon	1:100
TCRgd	PE – CF592	GL3	553178	BD Pharmingen	1:100
Live/ Dead-	Aqua 405		L34957	Thermo Fischer	1:1000
Buffer – BD Horizon™ Brilliant Stain Buffer			BD 566349	BD Biosciences	
Liberase™ TL			05401020001	Roche	0,8 U/mL

Supplementary Table S3. Histological Reagents

Antibody/Reagent	Clone	Identifier	Source
Anti-CD3	SP7	ab16669	Abcam
Anti-CD3		790-4341	Roche/Ventana
Anti-CD45-R0		M0742	DAKO
Acti-stainTM 555 Phalloidin		PHDH1	Cytoskeleton
Isolectin GS-IB4 AFTM 568 Conjugate		I21412	Thermo Fischer Scientifc

Goat anti-Rabbit IgG (H+L) Highly Cross-Adsorbed Secondary Antibody, Alexa Fluor™ 488		A11034	Invitrogen
Goat anti-Mouse IgG (H+L) Highly Cross-Adsorbed Secondary Antibody, Alexa Fluor™ 568		A11031	Invitrogen
Alexa-Fluor® 647 -AffiniPure Donkey Anti-Rabbit IgG (H+L)		711-605-152	Dianova
DAPI (for murine stainings)		10236276001	Roche
DAPI (for human stainings)		62248	Thermo Scientific
Dako Flourescence Mounting Medium		S3032	Agilent Technologies
Paraformaldehyd 16% Aqueous solution EM grade		15710	Electron Microscopy Sciences

Supplementary Table S4. Genes and Primers for qPCR

**Commercially available primers** (all purchased at ThermoFischer Scientific)		
**Gene**	**Gene name**	**Assay ID**
C3	complement component 3	Mm00437858_m1
Cfh	complement component factor h	Mm01299248_m1
Ctla2a	cytotoxic T lymphocyte-associated protein 2 alpha	Mm00484032_g1
Foxp3	forkhead box P3	Mm00475162_m1
Icam1	intercellular adhesion molecule 1	Mm00516023_m1
Il10	interleukin 10	Mm01288386_m1
Il15	interleukin 15	Mm00434210_m1
Il17a	interleukin 17A	Mm00439618_m1
Il1b	interleukin 1 beta	Mm00434228_m1
Il2	interleukin 2	Mm00434256_m1
Il22	interleukin 22	Mm01226722_g1
Il23a	interleukin 23	Mm00518984_m1
Il6	interleukin 6	Mm00446190_m1
Il7	interleukin 7	Mm01295803_m1
Itgb2	integrin beta 2	Mm00434513_m1
Rbp3	retinol binding protein 3	Mm01220858_m1

**Designed primers** (all synthesized by Biotez, Berlin, Germany)		
**Gene**	**Gene name**	**Sequences**
18s	18s ribosomal 1	F: ACA TCC AAG GAA GGC AGC AG
		R: TTT TCG TCA CTA CCT CCC CG
		Probe: 6-FAM CGC GCA AAT
Ccl2	C-C motif chemokine ligand 2	F: GGC TCA GCC AGA TGC AGT TAA
		R: CCT ACT CAT TGG GAT CAT CTT GCT
		Probe: CCC CAC TCA CCT GCT GCT ACT CAT TCA
Ccl5	C-C motif chemokine ligand 5	F: GCA GTC GTG TTT GTCACT CGA A
		R: GAT GTA TTC TTG AAC CCA CTT CTT CTC
		Probe: FAM-AAC CGC CAA GTG TGT GCC AAC CC-TAMRA
Cxcl1	C-X-C motif chemokine ligand 1	F: CTG CAC CCA AAC CGA AGT C
		R: AGC TTC AGG GTC AAG GCA AG
Il4	interleukin 4	F: GGC ATT TTG AAC GAG GTC ACA
		R: GAC GTT TGG CAC ATC CAT CTC
Pgf	placental growth factor	F: TGG TGC CTT TCA ACG AAG TG
		R: CAT CCA AGA TGT ACA CCA GCT TCT
		Probe: FAM- TCG CAG CTA CTG TCG GCC CAT G
Tcrb	T cell receptor beta	F: GGC ACA ACT CTC GAA ACC A
		R: CCA CTT GTC CTC CTC TGA AAG C
		Probe: FAM-TCC CGC TGC CAA GTG CAG TTC CA-TAMRA
Tgfb1	transforming growth factor beta 1	F: CCC GAA GCG GAC TAC TAT GC
		R: TAG ATG GCG TTG TTG CGG T
Tnf	tumor necrosis factor	F: GGT CCC CAA AGG GAT GAG AA
		R: TGA GGG TCT GGG CCA TAG AA
		Probe: FAM-TTC CCA AAT GGC CTC CCT CTC ATC A-TAMRA
